# Conceptual assessment of HRQOL among Japanese non‐metastatic castration‐resistant prostate cancer (nmCRPC) patients

**DOI:** 10.1002/cam4.4955

**Published:** 2022-06-30

**Authors:** Kazuo Nishimura, Masaki Shiota, Masatoshi Eto, Takefumi Satoh, Angela Stroupe, Caroline Seo, Alyssa Uzumcu, Dianne Athene Ledesma

**Affiliations:** ^1^ Department of Urology Osaka International Cancer Institute Osaka Japan; ^2^ Department of Urology, Graduate School of Medical Sciences Kyushu University Kyushu Japan; ^3^ Satoh Takefumi Prostate Clinic Tokyo Japan; ^4^ Open Health Newton Massachusetts USA; ^5^ Market Access Oncology Bayer Yakuhin, Ltd. Tokyo Japan

**Keywords:** prostate cancer, CRPC, clinical outcomes assessment, patient‐reported outcomes

## Abstract

**Objective:**

The study objectives were to understand how patients view the quality of life in non‐metastatic castration‐resistant prostate cancer (nmCRPC), including unmet needs and what patients consider most important in treatment outcomes. A gap analysis was conducted on existing patient‐reported outcomes (PROs) measures versus what is missing from the patient perspective, to guide future development of PRO‐based real‐world evidence for nmCRPC in Japan. A conceptual model for nmCRPC Japanese patients’ HRQOL was also created.

**Methods:**

This non‐interventional, qualitative study consisted of a targeted literature review, PRO instrument review, and interviews with 20 nmCRPC patients and five treating physicians. Triangulation of the gap analysis, evidence from the targeted review of the literature, and qualitative interview findings were employed to assess the comprehensiveness of current nmCRPC and HRQOL measures.

**Results:**

Symptoms most reported by patients were frequent urination (70%), nocturia (65%), and general pain (65%). Others reported included lack of strength (30%). HRQOL impacts most reported were anxiety (45%) and worry (50%) about their diagnosis. Additional impacts mentioned were weight changes, loss of sleep, difficulty walking, loss of appetite, and difficulty traveling and seeking toilets in public.

The gap analysis revealed 31 symptoms and 33 impacts not covered in existing prostate cancer‐specific PRO instruments. Patients mentioned musculoskeletal symptoms such as fractures, leg pain, cramps, numbness, and loss of leg bone strength. Impacts not previously discussed in the literature or in outcome measures were feelings of self‐consciousness around diagnosis, stigma around illness, and the impact on mobility including traveling.

**Conclusion:**

Key results reveal pain and urinary symptoms are the most experienced by Japanese nmCRPC patients. The diagnosis and treatment of disease leads to significant impacts in patient lives. Analysis revealed that symptoms and life impacts are missing in the current literature and outcome measures. Testing and debriefing of specific items could further substantiate these dimensions.

## Graphical abstract

The study objectives were to understand how patients view quality of life in non‐metastatic castration resistant prostate cancer (nmCRPC), including, unmet needs, and what patients consider most impactful in treatment outcomes. Key results reveal pain and urinary symptoms are the most experienced by Japanese nmCRPC patients. The diagnosis and treatment of disease leads to significant impacts in patient lives. Analysis revealed that symptoms and life impacts are missing in the current literature and outcome measures. Testing and debriefing of specific items could further substantiate these dimensions.

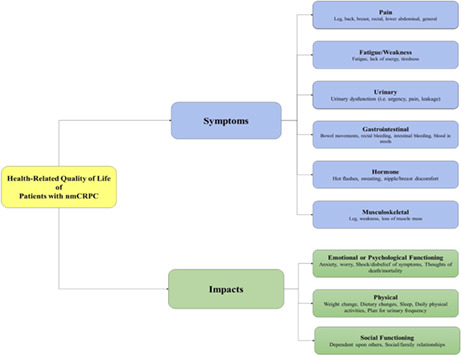

## 1 INTRODUCTION

Among Japanese men, prostate cancer is the 4th most common of all male cancers, with 78,400 new cases estimated to be diagnosed in 2018.^1^ Castration‐resistant prostate cancer (CRPC) develops in approximately 10%–20% of patients with prostate cancer within about 5 years after initial diagnosis. Of these patients, 84% will have metastases by the time CRPC is diagnosed. In 33% of those without metastases at CRPC diagnosis, bone metastasis will develop within 2 years.^2^

Compared with metastatic CRPC, non‐metastatic castration‐resistant prostate cancer (nmCRPC) has a more indolent natural history, with the M0 disease phenotype usually asymptomatic. Prognosis is determined by the patient's level of PSA elevation and PSA doubling time (PSADT), which together are used to predict whether metastasis will develop and when.^3^

While there is limited information about patients’ perceptions of nmCRPC symptoms and impacts, a recent study found that urinary, intestinal, and sexual symptoms were identified in the literature. Additionally, clinician interviews stressed that erectile dysfunction and loss of sexual desire were important impacts for nmCRPC patients, as were incontinence, urgency, and hot flashes. Furthermore, patients considered symptoms and effects that may be related to treatment as having more impact than the disease itself.^4^, ^5^

Symptoms and impacts can be ascertained through patient‐reported outcome (PRO) instruments. Prostate cancer‐related PRO instruments have been previously developed and are used in this field. However, whether these measures are appropriate for Japanese nmCRPC patients remains unclear. One systematic review of health‐related quality of life (HRQoL) instruments suggested that the HRQoL instruments most commonly used in this field were not designed specifically for an nmCRPC population. As a result, patients with localized rather than advanced diseases may be the more appropriate users of these instruments.^6^

Because there is limited patient‐centered data collected among the nmCRPC population in Japan, this study's objectives were to explore key concepts about nmCRPC that are important to Japanese clinicians and nmCRPC patients and assess whether current patient‐reported outcomes cover these key concepts.

## 2 METHODOLOGY

### 2.1 Study design

This non‐interventional, qualitative study consists of a targeted literature review and PRO instrument review, 20 patient and five physician face‐to‐face interviews, and a gap analysis to understand the unmeasured needs of Japanese nmCRPC patients.

The primary objective was to identify key concepts (treatment benefits/risks) important to nmCRPC patients in Japan. The secondary objective was to deliver a gap analysis on what is being measured in existing relevant PRO measures versus what else needs to be addressed from the patient perspective to guide the future development of PRO‐based real‐world evidence generation for CRPC.

While this study was exploratory in nature and the objectives were not to assess the usability of a current instrument or design a draft PRO instrument, this study's methodological approach and analysis were conducted according to PRO Good Research Practices Task Force Guidelines for Establishing Content Validity‐Eliciting Concepts for a New PRO Instrument Part 1 from the International Society for Pharmacoeconomics and Outcomes Research (ISPOR).^7^ A preliminary conceptual framework is provided to highlight the key symptoms and impacts that the patients interviewed experienced.

### 2.2 Targeted literature review and gap analysis

The targeted literature and instrument review were done to identify nmCRPC‐related symptom and impact concepts and better understand the humanistic burdens of nmCRPC. The primary search was conducted using ProQuest, with Embase and MEDLINE as the literature review sources. An additional search for relevant instruments with potential application in nmCRPC was conducted using PROQOLID to further assist in the identification of PRO measurement gaps. The literature search was not limited by dates or years, due to the lack of relevant literature available in this area. The search terms included variations on nmCRPC, HRQoL, PRO, and humanistic burden. Both free‐text and MeSH terms were used (see search strategy in Table S1). The eligibility of publications was assessed based on pre‐specified inclusion and exclusion criteria (Table S2) in 2 rounds:

1. Based on the review of the publication's title and abstract
2. Based on the full‐text review of publications that were not excluded in Round 1

Additionally, a PRO instrument gap analysis was performed to (1) identify concepts reported by Japanese nmCRPC patients that are not covered in the existing prostate cancer‐specific PRO instruments, and (2) determine if there are any existing prostate cancer‐specific PRO instruments that would be relevant and appropriate for use in the nmCRPC patient population.^8^, ^9^, ^10^ A preliminary conceptual framework to comprehensively describe nmCRPC health status was developed using frequently reported concepts emerging from both the targeted literature review and interviews, also referring to previously reported conceptual frameworks.^5^, ^11^

### 2.3 Patient and physician interviews

Patients were recruited from three institutions spread out into western, southern, and eastern Japan that were blinded per author guidelines. Treating clinicians screened and identified eligible patients. Bone scan, computed tomography (CT), and/or magnetic resonance imaging (MRI) were used to determine nmCRPC status. Once patients were found eligible, clinicians explained the study details and provided patients with an informational leaflet. Patients who were interested in being participants were consented and scheduled for a one‐on‐one interview lasting approximately 60 minutes on their next clinic visit.

Inclusion criteria for patients were: (1) Aged 20 years old or older, (2) Male, diagnosed with nmCRPC, (3) Eastern Cooperative Oncology Group‐Performance Status (ECOG‐PS) score of 0‐1. (4) Previously treated with either first‐generation anti‐androgen or novel anti‐hormones at least once, and (5) Could provide written informed consent. Patients were excluded based on whether they met any of these criteria: (1) They were participating in an investigational program that included interventions outside of what they would receive in routine clinical practice, (2) Diagnosis of metastasis to either bone or viscera through routine imaging, (3) A clinically relevant condition (medical or psychiatric) that could interfere with completing the study.

Board‐certified physicians were recruited through a market research panel, and none of the physicians were the recruited patients’ treating physicians. Physicians had to fulfill the following inclusion criteria: (1) They had to be a board‐certified urologist or oncologist, (2) They had to have treated at least three Japanese CRPC patients in routine clinical practice, (3) They must have administered first‐generation anti‐androgens or novel anti‐hormones in a clinical setting, and (4) They must have been able to provide written informed consent. There were no exclusion criteria for physicians.

The respective institutional review board (IRB) of each institution approved the conduct of this study. In the case where there was no in‐house IRB, approval came through a central IRB. All participants provided informed consent before study participation.

### 2.4 Interview methodology

The purpose of this phase was to document the concepts that are relevant and important to Japanese patients to conceptualize the symptom and treatment burden of nmCRPC.

Data collection was based on grounded theory methodology, specifically developed to use rigorous qualitative research to understand patients’ experiences with respect to treatments and treatment outcomes.^12^ The interviews were conducted in a stepwise manner, with data analyzed iteratively before a new round of interviews was conducted to allow the team to confirm that emerging themes and concepts were more clearly explained in subsequent interview rounds. In this fashion, patients’ input (grounded) was used to discern concepts, as opposed to applying and priori theoretical model to interpret the data. This allowed the voice of the patient to be heard.

The interviews were conducted face to face with Japanese nmCRPC patients, using semi‐structured qualitative interview guides for concept elicitation (CE). The interview guides were drafted based on the literature review and expert opinion input. In these CE interviews, qualitative researchers, all of whom were native speakers of Japanese, began with general exploratory questions and built upon them to ensure that intended themes were covered.

For this preliminary study, we framed our interview questions to inquire on patients’ self‐reported symptoms that they attributed to their nmCRPC. When conducting the interviews, probing was used to clarify patients’ attribution of symptom experiences. When analyzing the data, attribution codes were used to highlight when a patient was attributing a symptom or side effect to a specific treatment which delineated the symptoms patients associated specifically to their nmCRPC. Furthermore, data from the physician interviews and the study team's clinical expertise guided attribution of symptoms to either nmCRPC disease or treatment.

### 2.5 Analysis methodology

The process for coding qualitative interviews included an assessment of inter‐coder reliability by having the first two transcripts coded independently by each member of the qualitative team.^12^ Then, the team reviewed the coding together to ensure team consensus that the appropriate coding approach was achieved. Each transcript was dual‐coded to ensure a thorough review. When new codes arose, team members alerted one another through a shared discussion guide. In appropriate circumstances, the code was then defined and added to the coding scheme. Discussion on codes, coding, and the development of concepts proceeded throughout the qualitative data collection and analysis. The project director reviewed all coding to ensure that the codes were used in a consistent way across the team of coders and across transcripts. Codes were categorized via thematic analysis.

### 2.6 Saturation assessment

Saturation—the point at which further interviews do not lead to new concepts or issues in the nmCRPC experience—was assessed with a process that examines concepts across sets of consecutive interviews.^7^, ^12^, ^13^, ^14^ To assess saturation of concepts, patient transcripts were organized in chronological order, then divided into four groups with about five transcripts each. To evaluate saturation, the concepts that were derived from the second group of interviews were compared with concepts from the first group to determine if any additional information at the conceptual level emerged from interviews with the nmCRPC patients in the second group. If any new concepts were identified in the second group's transcripts, then saturation was not identified as having been achieved. For all consecutive groups, this comparison was repeated, and the study team determined saturation to have been met when no new themes emerged in the last set of interviews.

Saturation was not assessed in clinician interviews. Five practicing clinicians were deemed enough to provide the professional input needed.

## 3 RESULTS

### 3.1 Targeted literature review

**FIGURE 1:**
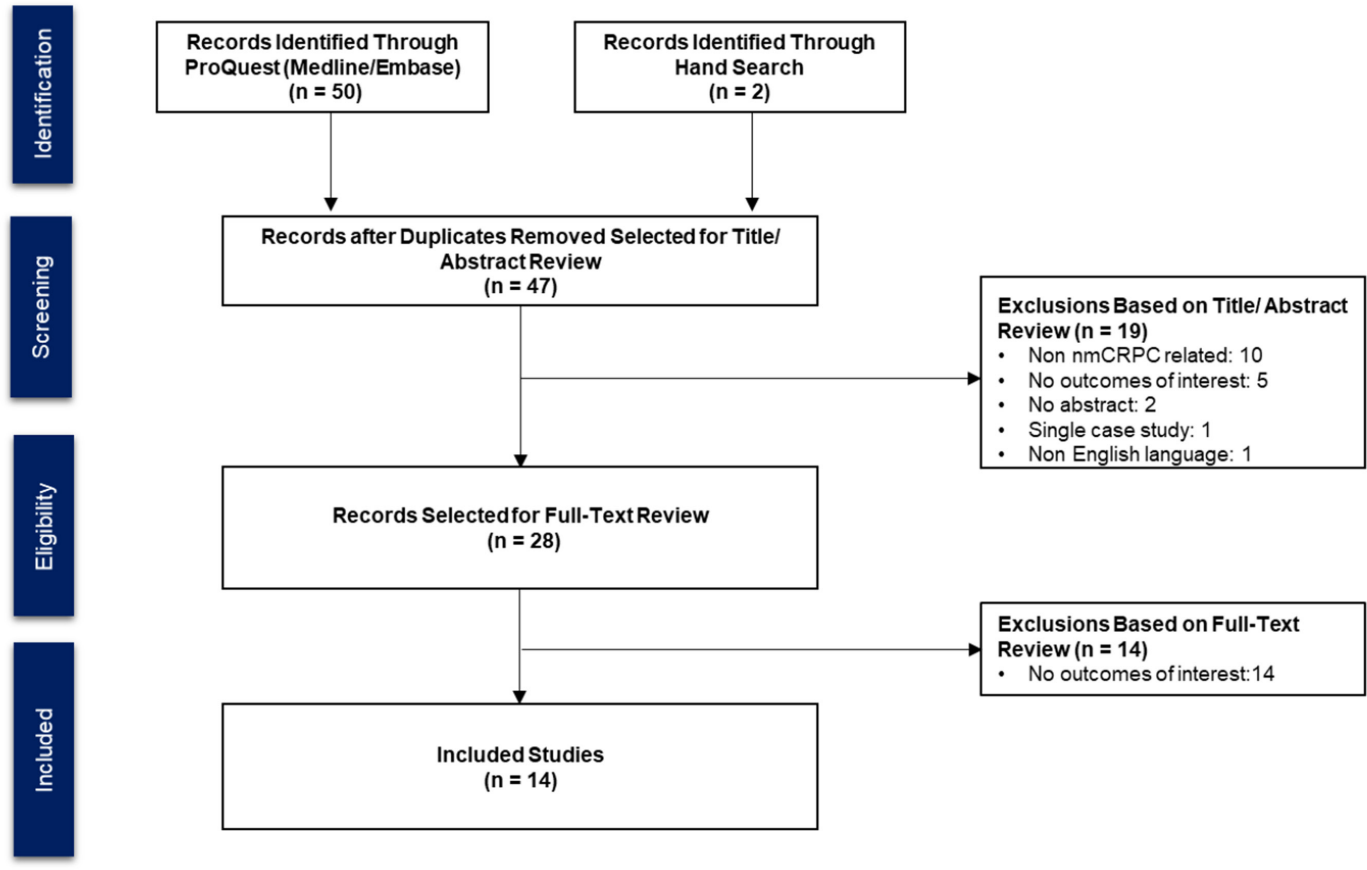
PRISMA diagram

The ProQuest literature search conducted on October 17, 2018 yielded 14 citations for inclusion (Table S3). Most of the articles or abstracts were excluded due to lack of outcomes of interest (*n* = 19) or not being related to the nmCRPC disease area (*n* = 10) (see Figure 1, PRISMA diagram). The FACT‐P was the most frequently used disease‐specific questionnaire to assess HRQoL in clinical trials for prostate cancer.

### 3.2 Identified concepts in the literature

Based on the literature review, 44 symptom concepts and 29 impact concepts were identified.

The symptom concepts can be categorized into the following major groupings (see Table 1A): Fatigue, gastrointestinal symptoms, general symptoms, hormonal symptoms, musculoskeletal symptoms, sexual function, urinary symptoms, and other symptoms.

**TABLE 1:** Symptom and impacts concepts identified from the literature review

A. Symptom concepts	Literature Review
**Fatigue** (fatigue, lack of energy, tired)	✓
**Gastrointestinal symptoms** (Bowel movement urgency during urination, Constipation, Cramp/pain in abdominal, Cramp/pain in pelvis, Cramp/pain in rectum, Frequent bowel movement, Radiation proctitis, Stool leakage	✓
**General symptoms** (Feel ill, Inability to lose weight, Pain from the waist down, Urge to eat, Weakness from the waist down, Weight gain, Weight loss	✓
**Hormonal symptoms** (Breast tenderness, Enlarged nipples/breast, Hot flashes/flushes)	✓
**Musculoskeletal symptoms** (Loss of muscle mass)	✓
**Sexual function** (Erectile dysfunction, Have/maintain erection, Loss of libido, Reach orgasm, Sexual desire)	✓
**Urinary symptoms** (Blood in urine, Burning with urination, Difficulty urinating, Doesn't feel urination, Groin pain, Incomplete emptying, Incontinence, Loss of penis length, Pain with urination, Painful ejaculation, Pressure/blockage while urinating, Urgency to pass urine/ urinary urgency, Urinate frequently, Urine leakage, Wake up to urinate, Weak urine stream)	✓
**Other** (Loss of hair)	✓

B. Impact concepts	
**Activity Limitation** (Interference with daily activities, Work)	✓
**Emotional/ Psychological functioning** (Anxiety/Anxious, Decreased coping ability, Depressed, Difficulty concentrating, Distrust in doctor, Embarrassed, Feel lonely, Feeling of loss of control, Frustration, Lack of confidence in doctor, Lose hope / hopelessness, Loss of masculinity, Low self‐esteem, Nervous, Thoughts on death and mortality, Worried of embarrassment if I try to have sex)	✓
**Gastrointestinal impacts** (Lacked appetite, Not absorbing nutrients, Plan for bowel movement)	✓
**Physical functioning** (Sleep)	✓
**Social functioning** (Become homebody, Become private, Stress/conflict with partner, friends, family, Unable to sustain relationship, Unable to sustain social life)	✓
**Urinary impacts** (Plan for urinary frequency)	✓
**Other impacts** (Financial difficulties)	✓

The impact concepts can be categorized into the following major groupings (see Table 1B): Activity limitation, emotional/psychological functioning, gastrointestinal impacts, physical functioning, social functioning, urinary impacts, and other impacts.

### 3.3 Patient characteristics

Twenty nmCRPC patients were recruited. Their median age was 76.5 years, and their median age at diagnosis was 63.5 (*N* = 18). The median time from prostate cancer (PC) diagnosis to nmCRPC diagnosis was 9.18 years (*N* = 18). At the time of enrollment, the median time from the most recent imaging procedure confirming non‐metastatic status was 3.73 months. In addition, 19 patients were classified as “asymptomatic” by their physicians, and 18 patients had an ECOG score of 0 (a score of 0 indicates the individual lives a fully active life and is able to carry on all pre‐disease activities without restrictions). Half of the patients were classified as either T3a or T3b at PC diagnosis (30%, 20% respectively), a majority of patients had no regional lymph node involvement (N = 85%), and no patients had metastasis (M0, 100%). Fifteen patients (75%) reported comorbidities; cardiovascular disease (30%) and hypertension (35%) were the most prevalent among the group. Patients’ detailed clinical characteristics and demographics are listed in Table 2.

**TABLE 2:** Patient clinical and demographic characteristics

Demographic variable	Category	Total sample (*N* = 20)
Age, median (range), years		76.5 (62–92)
Age at diagnosis, median (range), years		63.5 (54–80)^a^
Marital status, *n* (%)	Married	18 (90)
	Widowed	2 (10)
Education level, *n* (%)	Junior high school	2 (10)
	High school	7 (35)
	College	11 (55)
Employment status	Employed full‐time	1 (5)
	Self‐employed	4 (20)
	Retired	14 (70)
	Not employed	1 (5)
Area residence, *n* (%)	Kanto	5 (25)
	Kinki/Kansai	10 (50)
	Kyushu	5 (25)
Medical insurance, *n* (%)	National health insurance	5 (25)
	Late stage elderly insurance	13 (65)
	Other (company/social insurance)	2 (10)
Used high cost medical benefit system *n* (%)	Yes	8 (40)
Primary caregiver, *n* (%)	Yes^b^	7 (35)
Clinical variable	Category	Total sample (*N* = 20)
Time from PC to CRPC diagnosis, median (range), years		9.18 (1.36–18.45)^a^
Time from most recent imaging procedure confirming non‐metastatic status to enrollment date, Median (Range), months		3.73 (0.69–39.45)
Baseline tumor classification, *n* (%)	T	
	1	1 (5)
	2	6 (30)
	3a	6 (30)
	3b	4 (20)
	4	1 (5)
	Unknown	2 (10)
	N	
	N0	17 (85)
	N1	3 (15)
	M	
	M0	20 (100)
Symptomatic status at enrollment^c^, *n* (%)	Symptomatic	1 (5)
	Asymptomatic	19 (95)
ECOG performance status, *n* (%)	0	18 (90)
	1	2 (10)
Treatment initiated after PC diagnosis, *n* (%)	Active surveillance/watchful waiting	0 (0)
	Surgery (orchiectomy, prostatectomy)	2 (10)
	LHRH agonist/antagonist	20 (100)
	Anti‐Androgen	12 (60)
	Radiotherapy	9 (45)
	Chemotherapy	0 (0)
Treatment continued at time of CRPC diagnosis, *n* (%)	Active surveillance/watchful waiting	1 (5)
	Surgery (orchiectomy, prostatectomy)	0 (0)
	LHRH agonist/antagonist	17 (85)
	Anti‐Androgen	12 (60)
	Chemotherapy	0 (0)
	Radiotherapy	0 (0)
Treatment initiated after CRPC diagnosis, *n* (%)	Active surveillance/watchful waiting	0 (0)
	Surgery (orchiectomy, prostatectomy)	0 (0)
	LHRH Agonist/antagonist	10 (50)
	Anti‐Androgen	12 (60)
	Radiotherapy	0 (0)
	Chemotherapy	2 (10)
	Other^d^	5 (25)
Current treatments, *n* (%)	Active surveillance/watchful waiting	0 (0)
	Surgery (orchiectomy, prostatectomy)	0 (0)
	LHRH agonist/antagonist	17 (85)
	Anti‐Androgen	7 (35)
	Radiotherapy	0 (0)
	Chemotherapy	1 (5)
	Estrogens/progesterone	1 (5)
	Other^d^	7 (35)
Co‐Morbidities, *n* (%)	None reported	5 (25)
	Cardiovascular disease	6 (30)
	Chronic pulmonary disease	2 (10)
	Mild liver disease	2 (10)
	Diabetes without chronic complications	5 (25)
	Renal disease	4 (20)
	Hypertension	7 (35)
	Prior malignancy, now in remission (malignancy other than that of the prostate)	3 (15)

Abbreviations: ADT, Androgen Deprivation Therapy; LHRH, Luteinizing Hormone Releasing Hormone.

^a^ Two patients’ date of PC and CRPC diagnoses were unknown.

^b^ Wife was the only primary caregiver reported by the patients.

^c^ “Symptomatic” is defined as either regular (not occasional) use of analgesic medication or opioids for cancer related bone pain {(≥ level 1; World Health Organization [WHO] ladder for cancer pain) or requiring treatment with external beam radiation therapy (EBRT) for bone pain (the EBRT should be within previous 12 weeks)}.

^d^ Other treatments reported: 1: Chlormadinone acetate, 2: Enzalutamide, Apalutamide.

#### 3.3.1 Patient‐reported signs & symptoms

##### 3.3.1.1 Biomarker‐related signs

Nearly all patients expressed that high PSA levels (90%) led to their prostate cancer diagnosis and expressed how the elevated level was the only sign of prostate cancer. Fifteen patients (75%) reported an increase in PSA level over the course of their illness up to their current nmCRPC status. (Patients’ actual PSA values were not obtained in this study.)

Specifically, some patients described an increase in PSA as a reason for continued monitoring. One stated,

*“The doctor did say that it [PSA level] has been rising. He doesn*'*t think it*'*s a problem yet, but he wants to keep an eye on it, without changing anything yet.”* Another said about receiving or changing treatment, “*Yes, [the doctor] has changed the medication several times. I have tried all sorts; expensive drug, new drug, etc. when the PSA level started to rise.”*

##### 3.3.1.2 Symptoms

Table 3 presents the frequency and comprehensive symptom concepts reported by patients.

**TABLE 3:** Frequency of symptom concepts reported by Japanese nmCRPC patients

Concepts	Frequency, *n* (%)	Concepts	Frequency, *n* (%)
**General**		Swelling in legs or ankles	2 (10)
Bleeding	1 (5)	Loss of bone strength	1 (5)
Fever	1 (5)	Loss of muscles	3 (15)
**Pain (general)**	**13 (65)**	**Hematology**	
**Fatigue/Weakness**		Anemia	1 (5)
Fatigue	4 (20)	**Cardiology**	
Feel out of it	1 (5)	Aortic aneurysm	1 (5)
Feel sluggish/Lethargic	2 (10)	Arrhythmia	1 (5)
**Lack of strength/Feel weak**	**6 (30)**	**Digestive/ Gastrointestinal**	
Lack of energy	2 (10)	Blood in stools	1 (5)
Tiredness	4 (20)	Constipation	5 (25)
**Urology/Urinary**		**Diarrhea/loose stools**	**7 (35)**
Blood in urine	5 (25)	Hemorrhoid/intestinal bleeding	1 (5)
Blood in semen	1 (5)	Pain in lower abdomen	1 (5)
Difficulty stopping urine stream	1 (5)	Rectal bleeding	2 (10)
Difficulty urinating	5 (25)	Rectal area pain	1 (5)
Enlarged prostate	3 (15)	**Hormonal**	
**Frequent urination**	**14 (70)**	Breast pain	1 (5)
Hard areas on prostate	1 (5)	**Hot flashes/flushes**	**7 (35)**
Narrow urethra	2 (10)	Nipple pain/Nipple discomfort	4 (20)
**Nocturia**	**13 (65)**	Swelling in breast	5 (25)
Painful urination	3 (15)	**Other**	
Residual urine	3 (15)	Discomfort with face	1 (5)
Urgency to urinate	3 (15)	Bloated face	1 (5)
**Urine leakage/Urinary incontinence**	**7 (35)**	Bloating	2 (10)
**Weak/interrupted flow**	**9 (45)**	Dry sinus	1 (5)
**Musculoskeletal**		Face feelings burning	1 (5)
Bone fractures	3 (15)	Face feels hot	1 (5)
Leg cramp	2 (10)	Hair loss	5 (25)
Leg feel hot	1 (5)	Heavy feeling	2 (10)
Leg numbness	1 (5)	Inadequate erection	1 (5)
Leg pain	2 (10)	Polyp	1 (5)
**Leg weakness**	**6 (30)**	Sexual functioning	4 (20)

Symptoms reported by at least 30% of the patients interviewed are in bold; Nine(9) out of the 13 patients who reported General Pain also reported body area‐specific pain.

###### 3.3.1.2.1 Urinary‐related symptoms

Urinary‐related symptoms were reported by all patients, with urinary frequency (70%) and nocturia (65%) being the most common. Patients said that urinary symptoms were most burdensome and often hindrances to their daily activities and lives. One patient expressed the disruption of his commute, sharing,

“*I had to go to the toilet more frequently… when I would take the train, I would suddenly experience the urge to go to the toilet, and would have to get off at the next station, use the toilet, and get back on the train again to come to the hospital.”* Another patient expressed disruption in doing leisure activities, stating, *“For example, when I was playing golf, I needed to go to the toilet after playing every hole.”*

Urinary‐related issues also commonly disrupted patients’ ability to sleep well at night. A patient spoke of the struggle to obtain restful sleep, explaining,

*“[Getting up] twice is bearable. But when it*'*s up to 3 to 4 times during the night, it*'*s more difficult to get back to sleep, I*'*m awake longer and I get sleepy during the day.”* Another patient shared his loss of sleep, saying, *“During the night, after I woke up to go to the toilet and got back into bed and tried to fall back asleep, often, I would almost immediately have to go to the toilet again.”*

###### 3.3.1.2.2 Pain‐related symptoms

Sixty‐five percent (65%) of patients reported general pain symptoms. Specific areas of pain mentioned by these patients included nipple pain/discomfort (20%), painful urination (15%), leg pain (10%), lower abdomen (5%), rectal area (5%), and breast pain (5%).

Some patients reported symptoms of pain in relation to urinary issues. One patient attributed his painful urination to a narrowing of his urethra: *“It was sometimes painful when I was trying to urinate. I think the urethra had become very narrow which caused the pain, and I think there was some blood mixed in with the urine.”*

Another patient expressed that his pain stemmed from treatment‐related circumstances:

*I once asked the doctor, “I would like to go on a trip but because of this catheter, I am unable to go anywhere. I can't even go to the hot springs. Is there any way the catheter can be removed?” He suggested we could try, but then I am not sure whether the urine hardened up, but it became impossible for me to urinate. For 3 days I was in pain and I finally ran back to the doctor complaining of the pain and had the catheter reinserted. Forget about going on a trip, I decided I would never remove the catheter again—the pain was unbearable.*

###### 3.3.1.2.3 Fatigue/weakness

Patients’ experiences with fatigue were shared in terms of losing strength and the occurrence of weakness (30%), fatigue (20%), or tiredness (20%). Loss of strength and weakness were commonly experienced in patients’ legs. One patient said, “*Just a general feeling of lethargy*.” Other patients described weakness; one patient said,

“*My legs are not too strong anymore… I don*'*t remember when they got as weak as they are now. I am almost embarrassed when I walk now, it*'*s a choppy walk. And I*'*ve become very slow*.” Pain contributed to feelings of weakness; a patient describes the change in his leg strength, explaining, “*I did notice that my legs were getting weaker with age. And I had broken my leg after I fell. I also started to have some issues with my hip. I should have taken it easy, of course, but I had so many things to do.”*

###### 3.3.1.2.4 Gastrointestinal

The most reported symptom was diarrhea/loose stools (35%). Furthermore, some patients reported rectal bleeding (10%), hemorrhoidal bleeding (5%), or blood in their stool (5%). Some patients attributed the bleeding to localized radiation therapy, which contributed to some patients’ reporting rectal bleeding and blood in their stool. This experience coincided with pain in the rectal area; while temporary, this was a shocking experience. Said one patient,

*“I felt pain in my rectum, and I was bleeding. Rectal bleeding—I was shocked. The toilet bowl was bright red, and the blood wouldn*'*t stop. When I went to the hospital, they told me that it was proctitis caused by radiation therapy and they asked me that I had to be patient.”* Another patient surprised by his experience of bleeding shared, “*It happened all of a sudden. There were no symptoms or signs at all. We were shocked and called the ambulance, and then we were told that it was diverticulum bleeding*.”

###### 3.3.1.2.5 Hormonal

Hormonal changes were commonly experienced after androgen deprivation therapy (ADT) and patients acknowledged temporary symptoms of hot flashes (35%), nipple pain (20%), and breast swelling (25%). A patient who experienced a multitude of hormonal shifts shared his experiences, stating,

*Yes, you feel hot. I also experienced swelling in my breasts. In other words, prostate cancer feeds on male hormones so it is important that you suppress male hormones. Men also have female hormones. I experienced hormonal imbalance and as a result, felt hot flashes and sweated a lot. That continued for about half a year and disappeared with time.*

###### 3.3.1.2.6 Musculoskeletal

Leg weakness (30%), muscle mass loss (15%), and bone fractures (15%) were most reported by the patients. Loss of bone strength was also reported. Describing the impacts weakness and muscle loss can have, a patient explained his experience and the impact on his mobility, “*I felt like my bones were becoming weaker. When I was climbing onto my scooter one time, the scooter fell, and I broke 4 ribs… I really felt like my bones were becoming weaker.”*

#### 3.3.2 Patient‐reported impacts of living with nmCRPC

Table 4 summarizes the impacts reported by patients.

**TABLE 4:** Frequency of impact concepts reported by Japanese nmCRPC patients

Concepts	Frequency, *n* (%)	Concepts	Frequency, *n* (%)
**Emotional Functioning**		**Difficulty sleeping**	**7 (35)**
**Anxiety**	**10 (50)**	Difficulty traveling	2 (10)
Accepts diagnosis	4 (20)	**Difficulty walking**	**8 (40)**
Afraid of cancer	1 (5)	Increase in appetite	1 (5)
**Concerned**	**7 (35)**	Loss of appetite	3 (15)
Depression	2 (10)	**Loss of sleep**	**10 (50)**
**Diagnosis shock/surprise**	**13 (65)**	**Plan for urinary frequency**	**8 (40)**
Disappointed	1 (5)	Physical functioning	2 (10)
Disbelief at diagnosis	2 (10)	Seeking toilet	2 (10)
Embarrassed	3 (15)	Unable to travel	1 (5)
Feel beaten up/defeated	1 (5)	**Activity Limitation**	
Frustration	3 (15)	Interference in daily activities	4 (20)
Hesitant to go out	2 (10)	Dependent on others	2 (10)
Irritable	1 (5)	Inability to work	1 (5)
Lack of hope	2 (10)	Lifestyle changes	5 (25)
No will to keep going	1 (5)	Loss of jobs or business relationships	2 (10)
Patient dissatisfaction (i.e., treatment, care, doctor, etc.)	3 (15)	Coping with burden of nmCRPC (unknown expectations)	4 (20)
Planning for future	5 (25)	**Coping (adult diapers/incontinence pads)**	**9 (45)**
**Regret**	**6 (30)**	Coping (nipple pads)	1 (5)
Self‐conscious	3 (15)	Urinary catheter	1 (5)
Shock at symptom	1 (5)	**Social/Personal Relationships**	
Stigma around the illness	2 (10)	Personal/family relationships	3 (15)
Thoughts of death or dying	4 (20)	Social relationships	2 (10)
**Worry**	**13 (65)**	**Other**	
**Physical functioning**		Commuting burden	4 (20)
**Change in diet**	**8 (40)**	**Financial burden**	**7 (35)**
**Changes in weight**	**12 (60)**	Increased treatment cost due to changed treatment frequency	1 (5)
**Weight loss**	**7 (35)**	**Lack of physician communication**	**9 (45)**
Weight gain	5 (25)	Travel to treatment burden	2 (10)

*Note*: Impacts reported by at least 30% of the patients interviewed are in bold.

##### 3.3.2.1 Emotional/psychological functioning

Patients in this study expressed their concern about the future of their disease progression, general anxiety over their diagnosis, or their lack of informational resources and/or knowledge of available treatments (50%). One patient expressed the impact of receiving his cancer diagnosis eliciting general anxiety and the subsequent physical manifestation of the unknown in the form of night sweats while sleeping, stating,

*I can't explain very well, but it's almost like getting hit, or hitting something and I think, “Oh no!” and wake up. It's just a feeling, you know, of something going terribly wrong. I've experienced night sweats also. I'm not conscious of it, but I know it happened. It's hard to explain. It's not a specific worry or concern, but just a general feeling of anxiety that's all wrapped up and it comes out when I'm asleep.*

Regarding information sharing with their doctors, patients had opposing concerns: some expressed that the lack of treatment information made them feel anxious, while others said that the abundance of treatment or disease information made them feel anxious.

Lack of information:

*The doctor has tried three different therapies so far depending on the increase/decrease of the PSA level. He would say, ‘let's try this therapy next.’ He didn't really explain any details to me about the changes. This made me feel some mental anxiety because he didn't answer my question when I asked him for more explanation.”*

A lot of information:

*Although if he [the doctor] explains the [treatment] risks in detail, it can cause anxiety.*

##### 3.3.2.2 Physical functioning

Issues of pain, mobility issues, and/or hormonal treatment side effects that altered patients’ bodies, moods, and spirits ultimately had impacts on their daily lives. Patients experienced changes in their weight (60%) and diets (40%) due to cancer and associated treatments. In one case, a patient reported the effect of hormone treatment on his stomach's physical appearance, claiming,

*My tummy doesn't usually protrude, although I don't know if that happens with age, nor do I know whether it's a side effect of the medication. My doctor did say that there is a chance that I would see a change in my stomach area, and he did advise that I shouldn't eat until I get full, and that I should exercise.*

Appetite shifts were a way some of the patients were managing their cancer. Patients shared that they either intentionally changed their diet (e.g., ate less red meat, reduced salt intake) or they experienced appetite shifts—they were eating less as time went on. One patient explained, “*I still liked to eat the same things, but in general, I couldn*'*t eat as much food. I just got used to not eating as much, and now I*'*m eating less.”*

##### 3.3.2.3 Social/personal relationships

Patients reported changes in personal/family relationships (15%), as well as social relationships (10%) due to nmCRPC. When reflecting upon the potential for disease progression, one patient expressed his concern for how this could impact his relationship with his spouse, explaining, “*I just hope I do not become a burden on my wife.”*

### 3.4 Physician interviews

Participating urologists were male (100%), had a median age of 56 years old, and had an average of 26.8 years of clinical practice experience. They had treated an average of 9.8 nmCRPC patients and 18.6 CRPC patients in the past 3 months. As part of the inclusion criteria, all urologists had administered first‐generation anti‐androgens or novel anti‐hormones in a clinical setting.

When discussing nmCRPC symptoms, the five physicians participating in this study did not attribute symptoms to prostate can rather to the treatments that patients were enduring. For example, regarding frequent urination or urgency, one clinician attributed it to radiation or prostate surgery:

*[Urinary frequency] would not be directly associated with prostate cancer; however, some patients experience urinary frequency because of radiation therapy. Other patients may experience urinary incontinence or leaking urine after a surgery. Urinary disorder may appear as a side effect of cancer treatments so this may be one concern.*

Other non‐urinary symptoms physicians acknowledged being important to patients were loss of libido, fatigue, some pain (e.g., nipple pain and joint pain), and hot flashes, all of which were attributed to treatment side effects.

The primary HRQoL impacts that physician deemed most important to patients were the initial anxiety of the cancer diagnosis and depression, which they mostly attributed to the initial diagnosis or to adverse treatment effects. Explained one physician,

*Patients may experience temporary depression after hearing that they have cancer. However, most patients are able to manage the situation well and these psychological symptoms become alleviated with time. One of the adverse effects of hormone therapy is depression so some patients may feel down because of the treatment's adverse effect. However, that would not be frequent.*

Another physician reported that some patients experience the side effect of muscle loss as having an impact on their daily physical activities. He said, “*They have undergone hormone therapy so naturally, they must have experienced loss of muscles. They complain that they cannot enjoy sports and other activities/hobbies like they used to.”*

### 3.5 Preliminary conceptual framework

**FIGURE 2:**
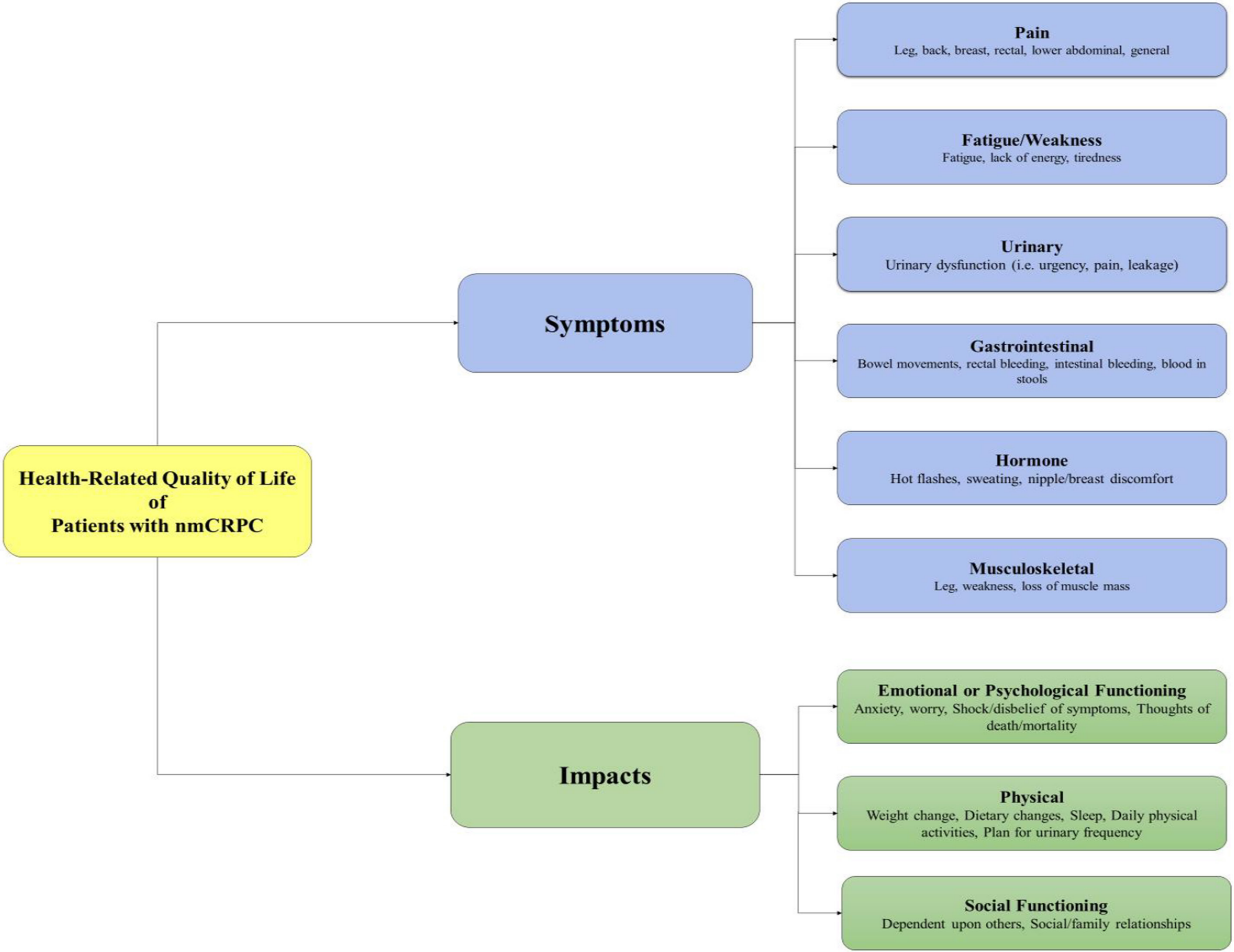
Preliminary conceptual framework for Japanese nmCRPC patients

As part of this study's objective was to understand the HRQOL of Japanese patients with nmCRPC, themes explored in this study's patient population were compared with concepts and themes presented in the literature, as well as with physician insights. Themes were mapped in a preliminary conceptual framework to highlight concepts most commonly reported by Japanese nmCRPC patients (see Figure 2). A model of the concepts can highlight key important features of the interviewed patient population.^7^ More importantly, they give us an idea of Japanese nmCRPC patients’ thoughts on HRQOL. The symptoms were broken into six symptom domains and three impact domains. Symptoms and impacts included were those most frequently reported in patient interviews and those most prevalent in the literature.

### 3.6 PRO instrument gap analysis

Following ISPOR's Good Clinical Practice guidelines,^7^ the gap analysis sought to compare concepts in current prostate cancer instruments like the EORTC QLQ‐PR25 and the FACT‐P with the data results from the literature review and patient interviews.^15^, ^16^, ^17^

Table 5 shows some symptoms/impacts reported by Japanese nmCRPC patients that are not currently covered by existing PRO scales. More details of the gap analysis of the symptom and impact concepts can be found in Tables S4 and S5, respectively. The concepts that were reported by patients but that are not covered in any of the existing prostate cancer‐specific PRO instruments include:

**TABLE 5:** Gap analysis of symptoms and impacts (Excerpts)

	Literature review	Present in any prostate cancer‐Specific PRO Instruments	Reported by physicians	Reported by patients
A. Symptom concepts				
Fatigue				
Fatigue	✓	✓	✓	✓
Feel out of it				✓
Feel sluggish/lethargic				✓
Feel weak/lack of strength		✓	✓	✓
Lack of energy	✓	✓		✓
Need to rest		✓		
Tired	✓	✓		✓
Musculoskeletal symptoms				
Bone fractures				✓
Bone pain		✓		
Leg cramp				✓
Leg numbness				✓
Leg pain				✓
Legs feel hot				✓
Loss of bone strength				✓
B. Impact concepts				
Activity limitation				
Daily activities		✓	✓	✓
Dependent on others				✓
Dressing self		✓		
Eating		✓		
Hobbies		✓		
Leisure activities		✓		
Lifestyle changes				✓
Loss of jobs or business relationships				✓
Hesitant to go out				✓
Difficulty traveling				✓
Unable to travel				✓
Commuting burden (public transportation)				✓
Plan for urinary frequency	✓			✓

• Symptoms (31 concepts): Hemorrhoids, increase in appetite, intestinal bleeding, rectal bleeding, bloated face, discomfort with face, dry sinus, face feeling burning/hot, heavy feeling, polyp, thirst, breast pain, nipple/pain discomfort, bone fractures, leg cramps, leg numbness, leg pain, legs feel hot, loss of bone strength, sexual functioning (general), blood in semen, difficulty stopping urine stream, hard areas on prostate, narrow urethra, residual urine, anemia, aortic aneurysm, arrhythmia, presence of mass, neurological disorder, and rash.

• Impacts (33 concepts): Dependent on others, lifestyle changes, loss of jobs or business relationships, afraid of cancer, concerned, diagnosis shock/surprise, disappointed, disbelief at diagnosis, doctor and patient communication, feeling beaten up/defeated, hesitant to go out, no will to keep going, patient dissatisfaction (i.e., treatment, care, doctor, etc.), planning for future, plan for urinary frequency, regret, self‐conscious, shock at symptom, stigma around the illness, change in diet, difficulty traveling, loss of sleep, physical functioning (general), unable to travel, personal/family relationships, social relationships, commuting burden, increased treatment cost due to changed treatment frequency, stress (general), the concern of treatment effectiveness, family concern over illness, hospital admittance, and travel to treatment burden

From our gap analysis, the most commonly reported urinary symptoms by Japanese nmCRPC patients were more frequently captured in the EORTC QLQ‐PR25 and the EPIC scales. General pain is captured both in the FACT‐P and EORTC QLQ‐PR25, but they do not capture specific pain experienced by this nmCRPC population such as leg, back, breast, abdominal, and rectal pain. Loss of muscle mass was represented in the literature review and reported by one physician, and leg weakness was reported by patient participants (30%); however, neither of these reported symptoms are captured in the FACT‐P or EORTC QLQ‐PR25.^18^ Symptoms related to hormonal treatments attributed to patients’ lived experience are captured by the EORTC QLQ‐PR25, although these attributes relating to patients’ feelings of well‐being and masculinity could be further explored as patient participants in this study expressed feelings of embarrassment due to nmCRPC. Finally, as seen in Tables S3 and S4, items on sexual functioning are variably included in either symptom or impact concepts in current PROs. In our study, patients reported more on sexual function symptoms, hence our categorization of these under “Symptoms.”

## 4 DISCUSSION

Even though nmCRPC is thought of as asymptomatic, this study's key results uncovered emerging symptoms and quality‐of‐life impacts that are intrinsic to the Japanese nmCRPC patient experience. Accurate assessments of a patient's disease experience including the humanistic burden and clinical and treatment experiences, as well as the symptoms and HRQoL impacts, can aid in improving clinical experiences. Discussing treatment decisions, supporting emotional health, and understanding unmet needs also increase the quality of health care a patient receives.

Japanese nmCRPC patients in this study mentioned several symptoms related to urinary dysfunction (frequent urination and nocturia being the most common), pain, fatigue, hormonal experiences, and musculoskeletal issues. These results are in agreement with previously reported nmCRPC symptoms from patients in the US, specifically urinary symptoms, fatigue, and hormonal experiences.^5^ They are also similar to the results of an international study that reported on symptoms experienced by advanced prostate cancer patients, specifically fatigue and urinary symptoms.^6^

The importance of recognizing urinary symptoms as impacts of PROs in nmCRPC is emphasized in this study. Urinary symptoms (including urinary frequency, incontinence, nocturia, and dysuria) have been reported to cause poor QoL, anxiety, depression, sleep and activity disturbance, and disruption of social activities.^19^, ^20^ As these symptoms were the most frequently reported, the need exists for effective interventions (clinical or otherwise) that could alleviate their occurrence in Japanese nmCRPC patients.

Pain is reported to be prevalent in men with CRPC, and the control of pain is considered an important therapeutic objective in CRPC because pain can be debilitating for patients. Hence treatment options demonstrating efficacy against cancer‐related pain should be given consideration in this population.^21^ Recent clinical trials in nmCRPC have shown different treatment options, with some nmCRPC treatments demonstrating efficacy in delaying time until pain progression.^22^

Data from the gap analysis show that there are many concepts expressed by the Japanese nmCRPC patients that are not covered in current PRO scales. However, due to some gap items being identified by only one or two patients, there may be a need to further explore whether these items are relevant to a broader Japanese nmCRPC or CRPC population to better understand patients’ HRQOL status. Still, the International Consortium for Health Outcomes has acknowledged that no single instrument adequately covers priority domains in HRQOL in CRPC, and a combination of different PRO instruments is recommended to capture these outcomes fully.^21^

Some emerging concepts that have yet to be fully explored deserve attention, such as musculoskeletal issues (leg cramps, leg numbness, leg pain) and bleeding. Some of these reported gaps, such as leg numbness, have also been reported elsewhere in literature as novel concepts not captured within existing prostate cancer measures.^23^

The impacts most prevalent in this study population were the feelings of shock/surprise at diagnosis (65%), worry (65%) and anxiety (50%) about disease progression. Because the feelings of shock/surprise were reported by patients in relation to their initial PC diagnosis, these may not be relevant to the nmCRPC state. However, worry and anxiety have been reported in other literature as part of the important impacts of advanced PC, hence careful attention should continue to be given to these impacts at this stage of the patient's disease, especially in terms of communication about treatment options and disease progression.^5^, ^24^

Based on the key findings from 20 patient interviews, five physician interviews, literature review results, and an analysis of these findings in comparison with the current prostate cancer‐specific measurements, a preliminary conceptual framework was developed. Although the nmCRPC state is generally thought of as asymptomatic, this framework emphasizes symptoms and impacts that we believe are important features to consider when assessing a Japanese nmCRPC patient's quality of life. This is the first conceptual model for Japanese nmCRPC patients that we are aware of and could serve as a guide for Japanese physicians in assessing their patients’ quality of life. Furthermore, this framework builds on other evidence on the nmCRPC quality‐of‐life concept presented elsewhere.^5^, ^11^ Still, more exploratory debriefing of these experiences could be conducted to ensure current measures are fit for purpose in nmCRPC clinical trial settings, as well as for assessments of patients’ quality of life in clinical practice.

### 4.1 Study limitations

The limitation of this study is the homogenous nature of the patient population that aided in early saturation of the concepts, although the novel concepts that emerged from the interviews were expressed by fewer than 50% of patients. Diversity of the data set was limited to TNM Classification at PC Diagnosis, treatment initiated after PC diagnosis, age, and geographic residence. Furthermore, only patients who could complete a 60‐minute interview were selected to participate in this study. Hence frailer patients or patients with ongoing mental difficulties for whom important nmCRPC concepts could have differed greatly from those from the current sample were excluded from this study.

Additionally, this qualitative study relies upon the patients’ perspectives of what the attribution of their symptoms were. The context of the questions were asked in relation to the patients’ experience with their nmCRPC, but also their experiences with hormone replacement therapy and if/when it wasn't clear what the patient was attributing the symptom too, the interviewer would often as a follow‐up question to clarify the symptom's attribution. Patients’ attribution of their symptoms and side effects due to their nmCRPC journey were coded to ensure when patients were clearly stating a side effect that it was attributed to a treatment therapy (i.e., Hormone replacement therapy). On the other hand, our own clinical experience also guided the analysis in grouping patients’ symptoms as to their relevance to nmCRPC or its treatments

Finally, some patients may have been reluctant to express their negative feelings and physical symptoms, hence there is a possibility that we were not able to fully explore all symptoms and impacts due to the patients’ reluctance to express these experiences.

Further qualitative assessment is recommended to support the value of the novel symptoms and impacts that are presented in this report.

## 5 CONCLUSIONS

The triangulation of the gap analysis, evidence from the targeted literature review, and qualitative interview findings were employed to determine the concepts that are most important to the nmCRPC population in Japan. The key results of this study reveal that urinary symptoms and pain are the symptoms most experienced by Japanese nmCRPC patients. Furthermore, our data show that assessing severity, degree of bother, and frequency of the symptoms and impacts would capture the entire continuum of Japanese nmCRPC patients’ lived experience. Additionally, considering field testing and debriefing of specific items found as gaps in this study are recommended, as these activities would further substantiate the development of a more disease‐specific measure.

### 5.1 Clinical implications

It is often reported that the nmCRPC state is an asymptomatic state, wherein patients do not experience considerable symptoms either as part of the disease continuum or as side effects of treatment. Our study shows that, to the contrary, nmCRPC patients do experience several symptoms that they consider bothersome enough to affect their quality of life. The goal of physicians is to understand that patients do experience these considerable symptoms, which should be considered thoroughly when making treatment decisions in nmCRPC and in communicating with their patients.

## AUTHOR CONTRIBUTIONS

Kazuo Nishimura contributed input to the study design as an expert opinion leader, as well as to data collection and the interpretation of the results. Masaki Shiota, Masatoshi Eto, and Takefumi Satoh contributed to data collection and the interpretation of study results. Angela Stroupe, Caroline Seo, and Alyssa Uzumcu created the study design and were responsible for data aggregation and analysis, as well as medical writing. Dianne Athene Ledesma contributed to the study design, data interpretation, and overall coordination of the study. All authors read and approved the final manuscript.

## FUNDING INFORMATION

This study was funded by Bayer Yakuhin, Ltd. Pharmerit International (now Open Health) received funding from Bayer Yakuhin, Ltd., for the conduct of the study and the development of the manuscript.

## CONFLICT OF INTEREST

Kazuo Nishimura declares the following conflicts of interest: Honoraria from speakers’ bureau (Astellas Pharma, Novartis, Bayer Yakuhin) and research grants (Bayer Yakuhin). Masaki Shiota declares the following conflicts of interest: Honoraria from speakers’ bureau (Janssen Pharmaceutical K.K., AstraZeneca K.K., Astellas Pharma) and a research grant (Daiichi Sankyo Company). Masatoshi Eto declares the following conflicts of interest: Honoraria from speakers’ bureau (ONO, Takeda, Novartis, Pfizer, BMS, Janssen, MSD, Merck) and scholarship donations (Sanofi, Bayer, Astellas, ONO, Takeda). Takefumi Satoh declares the following conflicts of interest: Honoraria from Janssen Pharmaceutical K.K., Bayer AG, Astellas Pharma, AstraZeneca, Nippon Shinyaku Co., Ltd. Angela Stroupe, Alyssa Uzumcu, and Caroline Seo declare the following conflict of interest: As employees of Pharmerit (now Open Health), they received consulting fees for study implementation, analysis, and publication from the study sponsor. Dianne Athene Ledesma declares the following conflict of interest: Full‐time employee of Bayer Yakuhin.

## ETHICS STATEMENT

The conduct of this study was approved by the respective institutional review board (IRB) of each participating institution, and through a central IRB where there was no in‐house IRB available. All study participants (physicians and patients) provided informed consent before study participation.

## Supporting information


Table S1:

Table S2:

Table S3:

Table S4:

Table S5:

## Data Availability

Availability of the data underlying this publication will be determined later according to Bayer’s commitment to the EFPIA/PhRMA “Principles for responsible clinical trial data sharing,” This pertains to scope, time point, and process of data access. As such, Bayer commits to sharing upon request from qualified scientific and medical researchers’ patient‐level clinical trial data, study‐level clinical trial data, and protocols from clinical trials in patients for medicines and indications approved in the United States (US) and European Union (EU) as necessary for conducting legitimate research. This applies to data on new medicines and indications that have been approved by the EU and US regulatory agencies on or after January 01, 2014. Interested researchers can use www.clinicalstudydatarequest.com to request access to anonymized patient‐level data and supporting documents from clinical studies to conduct further research that can help advance medical science or improve patient care. Information on the Bayer criteria for listing studies and other relevant information is provided in the study sponsors section of the portal. Data access will be granted to anonymized patient‐level data, protocols, and clinical study reports after approval by an independent scientific review panel. Bayer is not involved in the decisions made by the independent review panel. Bayer will take all necessary measures to ensure that patient privacy is safeguarded.
