# Supplementary material for: Conceptual assessment of HRQOL among Japanese non‐metastatic castration‐resistant prostate cancer (nmCRPC) patients
Source: Cancer Med. 2022 Jun 30;12(2):1762–78. doi: 10.1002/cam4.4955 (PMC9883429; doi:10.1002/cam4.4955)
Supplement: Supplementary file 1 — Table S1: [file CAM4-12-1762-s004.docx]

**Supplemental Table 1. Search Strategy for Target Literature Review**

| **Set#** | **Searched for** | **Results** |
| --- | --- | --- |
| S1 | TI,AB(“nmCRPC”) OR TI,AB(“nonmetastatic castration-resistant prostate cancer”) OR TI,AB(“non-metastatic castration resistant prostate cancer”) OR TI,AB(“nm-CRPC”) OR TI,AB(“nonmetastatic CRPC”) OR TI,AB(“non-metastatic CRPC”) OR TI,AB(“nonmetastatic castrate-resistant prostate cancer”) OR TI,AB(“M0 CRPC”) OR TI,AB(“M0 castration-resistant prostate cancer”) | 224 |
| S2 | (((TI,AB(burden) OR TI,AB("treatment burden") OR TI,AB("humanistic burden")) OR (TI,AB(burden NEAR/3 (caregiver OR family OR families OR society OR societal OR patient OR person))) OR (TI,AB(impact NEAR/3 (caregiver OR family OR families OR society OR societal or patient OR person))) OR ((TI,AB("unmet need"))))) | 536664 |
| S3 | TI,AB(care-giver*) OR TI,AB(caregiver*) OR TI,AB("quality of life") OR TI,AB("limitation of activity") OR TI,AB("patient reported") OR TI,AB("patient-reported") OR TI,AB(patient AND prefer*) OR TI,AB("patient satisfaction") OR TI,AB("patient participation") OR TI,AB("patient involvement") OR TI,AB("patient empowerment") OR TI,AB("patient engagement") OR TI,AB("patient activation") OR TI,AB(quality adjusted life) OR TI,AB(quality-adjusted life) OR TI,AB(qaly* OR qald*) OR TI,AB(“disability adjusted life”) OR TI,AB(“disability-adjusted life”) OR TI,AB(daly*) OR TI,AB(hql OR hqol OR h qol OR hrqol OR hr qol) OR TI,AB(health* AND year* AND equivalent*) OR TI,AB(health AND utility*) OR TI,AB(hui OR hui1 OR hui2 OR hui3) OR TI,AB(disutil*) OR TI,AB(“quality of well being” OR “quality of wellbeing” OR “quality of well-being” OR “qwb”) OR TI,AB(willingness to pay) OR TI,AB(standard gamble*) OR TI,AB(“time trade off” OR “time tradeoff” OR “tto”) OR TI,AB(index NEAR/2 well-being) OR TI,AB(quality NEAR/2 well being) OR TI,AB(health NEAR/3 utilit*) OR TI,AB(multi*attribute NEAR/3 health) OR TI,AB(multi*attribute NEAR/3 theor*) OR TI,AB(multi*attribute NEAR/3 health) OR TI,AB(multi*attribute NEAR/3 utilit*) OR TI,AB(multi*attribute NEAR/3 analys*) OR TI,AB(15D OR 15 dimension*) OR TI,AB(12D OR 12 dimension*) OR TI,AB(rating AND scal*) OR TI,AB(linear AND scal*) OR TI,AB(linear AND analog*) OR TI,AB(visual AND analog*) OR EMB.EXACT.EXPLODE (“quality of life”) OR EMB.EXACT.EXPLODE("quality of life assessment") OR EMB.EXACT.EXPLODE(“health status indicator”) OR MESH.EXACT (“quality of life”) OR MESH.EXACT.EXPLODE(“Health Surveys”) OR TI,AB(“functional status” OR “function” OR “functioning” OR “functional outcome” OR “function*” OR “functional assessment” OR “FSQ”) | 9137304 |
| S4 | ((((MESH(signs and symptoms))) OR ((EMB(signs and symptoms))) OR (TI,AB(symptom scale*) OR TI,AB(symptom assessment) OR TI,AB(social symptom*) OR TI,AB(assessment scale*) OR TI,AB(rating scale*) OR TI,AB(impact scale*)))) | 810964 |
| S5 | (S1 AND S2) | 21 |
| S6 | (S1 AND S3) | 31 |
| S7 | (S1 AND S4) | 9 |
| S8 | (S5 OR S6 OR S7) | 50 |
