# Supplementary material for: Conceptual assessment of HRQOL among Japanese non‐metastatic castration‐resistant prostate cancer (nmCRPC) patients
Source: Cancer Med. 2022 Jun 30;12(2):1762–78. doi: 10.1002/cam4.4955 (PMC9883429; doi:10.1002/cam4.4955)
Supplement: Supplementary file 2 — Table S2: [file CAM4-12-1762-s005.docx]

**Supplemental Table 2. Inclusion and Exclusion Criteria for Targeted Literature Review**

| **Inclusion Criteria** | **Exclusion Criteria** |
| --- | --- |
| - Studies involving adult patients - Randomized clinical trials - Observational studies - Retrospective studies - Qualitative studies - Studies in humans - Corrections to articles containing primary data - Articles will be restricted to English-only publications - Contains outcome measures incorporating any of the following aspects of humanistic burden: - General patient or caregiver health-related quality of life (HRQOL) - Instruments that have been used to measure HRQOL (e.g., content development, psychometric validity) - Symptoms and impacts | - Studies not specific to nmCRPC - Population ≤ 18 years of age - Non-English language studies (will include if abstract is in English) - No abstract available - Studies not in humans - Diagnostic studies - Single case studies/case-reports - Commentary/editorial/letter/non-systematic review - Secondary publications of studies reported elsewhere - Any pharmacological studies, pharmacokinetic, pharmacodynamics, and medicinal chemistry studies |
