# Supplementary material for: Conceptual assessment of HRQOL among Japanese non‐metastatic castration‐resistant prostate cancer (nmCRPC) patients
Source: Cancer Med. 2022 Jun 30;12(2):1762–78. doi: 10.1002/cam4.4955 (PMC9883429; doi:10.1002/cam4.4955)
Supplement: Supplementary file 3 — Table S3: [file CAM4-12-1762-s002.docx]

**Supplemental Table 3. PRISMA Table Literature Review References**

| **PRIMSA Table Literature Review References (N=14)** |
| --- |
| 1. **Dawson N, Rentz A, Lloyd A, et al. MP86-14 Patient valuation of castration-resistant prostate cancer health states. Journal of Urology. 2018;199(4S):e1183-e1183** 2. **Fizazi K, Shore ND, Tammela TLJ, et al. ARAMIS trial: Efficacy and safety phase 3 trial of ODM-201 in men with high-risk non-metastatic castration-resistant prostate cancer (nmCRPC). Journal of Clinical Oncology. 2015;33(15_suppl):TPS5080-TPS5080.** 3. **Heidenreich A, Sternberg C, Fizazi K, et al. P057 PROSPER: A phase 3 study of enzalutamide in non-metastatic (M0) castration-resistant prostate cancer (CRPC) patients. European Urology Supplements. 2014;13(5):130** 4. **Hussain M, Corn PG, Michaelson MD, et al. Safety, efficacy, and health-related quality of life (HRQoL) of the investigational single agent orteronel (ortl) in nonmetastatic castration-resistant prostate cancer (nmCRPC). Journal of Clinical Oncology. 2013;31(suppl; abstr 5076)** 5. **Hussain M, Fizazi K, Saad F, et al. PROSPER: A phase 3 study of enzalutamide in nonmetastaic (M0) castration-resistant prostate cancer (CRPC) patients. J Clin Oncol. 2014;32:5s(suppl; abstr TPS5094)** 6. **Hussain M, Fizazi K, Saad F, et al. Enzalutamide in men with nonmetastatic, castration-resistant prostate cancer. New England Journal of Medicine. 2018;378(26):2465-2474.** 7. **Lowrance WT, Murad MH, Oh WK, Jarrard DF, Resnick MJ, Cookson MS. Castration-resistant prostate cancer: AUA guideline amendment 2018. J Urol. 2018;200(6):1264-1272** 8. **Moïse P, Tomaszewski EL, Krupnick R, Baskin-Bey E, Meyer M, Holmstrom S. Identifying symptoms and impacts experienced by men with non-metastatic castration resistant prostate cancer. Value in Health. 2014;17(3):A94** 9. **Saad F, Cella D, Basch E, et al. Effect of apalutamide on health-related quality of life in patients with non-metastatic castration-resistant prostate cancer: an analysis of the SPARTAN randomised, placebo-controlled, phase 3 trial. Lancet Oncol. 2018;19(10):1404-1416** 10. **Saad F, Penson D, Attard G, et al. MP52-19 Impact of enzalutamide on pain and health-related quality of life in men with non-metastatic castration-resistant prostate cancer: PROSPER study results. Journal of Urology. 2018;199(4S):e703-e703** 11. **Saad F, Small E, Hadaschik B, et al. Patient (pt) reported outcomes (PROs) in SPARTAN, a phase 3, double-blind, randomized study of apalutamide (APA) plus androgen deprivation therapy (ADT) vs placebo (PBO) plus ADT in men with nonmetastatic castration-resistant prostate cancer (nmCRPC). European Urology Supplements. 2018;17(2):e1070-e1071** 12. **Smith MR, Saad F, Chowdhury S, et al. Apalutamide treatment and metastasis-free survival in prostate cancer. New England Journal of Medicine. 2018;378(15):1408-1418** 13. **Tomaszewski EL, Moise P, Krupnick RN, et al. Symptoms and impacts in non-metastatic castration-resistant prostate cancer: qualitative study findings. Patient. 2017;10(5):567-578** 14. **Tombal B, Hussain M, Penson D, et al. Patient-reported outcome measures in men with non-metastatic castration-resistant prostate cancer: baseline data from the PROSPER trial. Eur J Cancer. 2018;17(2):e870** |
