# Supplementary material for: Conceptual assessment of HRQOL among Japanese non‐metastatic castration‐resistant prostate cancer (nmCRPC) patients
Source: Cancer Med. 2022 Jun 30;12(2):1762–78. doi: 10.1002/cam4.4955 (PMC9883429; doi:10.1002/cam4.4955)
Supplement: Supplementary file 4 — Table S4: [file CAM4-12-1762-s003.docx]

**Supplemental Table 4. Gap Analysis for Japanese nmCRPC patients- Symptoms**

| **Concepts** | **Literature**  **Review** | **Presence in Prostate Cancer-Specific PRO Instruments** | | | | | | | **Physician Interviews** | **Patient Interviews** |
| --- | --- | --- | --- | --- | --- | --- | --- | --- | --- | --- |
|  |  | **FACT-G + P** | **EORTC QLQ-C30 + PR25** | **NCCN-FACT FPSI-17** | **PC-QoL** | **PROSQOLI** | **UCLA-PCI** | **EPIC** | (N=5),  n (%) | (N=20),  n (%) |
| **Fatigue** |  |  |  |  |  |  |  |  |  |  |
| Fatigue | ✓ |  |  | ✓ |  | ✓ |  |  | 1 (20) | 4 (20) |
| Feel out of it |  |  |  |  |  |  |  |  |  | 1 (5) |
| Feel sluggish/lethargic |  |  |  |  |  |  |  |  |  | 2 (10) |
| Feel weak/lack of strength |  |  | ✓ |  |  |  |  |  | 1 (20) | 6 (30) |
| Lack of energy | ✓ | ✓ |  | ✓ |  |  | ✓ | ✓ |  | 2 (10) |
| Need to rest |  |  | ✓ |  |  |  |  |  |  |  |
| Tired | ✓ |  | ✓ |  |  | ✓ |  |  |  | 4 (20) |
| **Gastrointestinal symptoms** |  |  |  |  |  |  |  |  |  |  |
| Appetite (general) |  |  |  |  |  | ✓ |  |  |  |  |
| Bloated |  |  | ✓ |  |  |  |  |  |  | 2 (10) |
| Blood in stool |  |  | ✓ |  |  |  |  | ✓ |  | 1 (5) |
| Bowel movements |  | ✓ |  | ✓ |  | ✓ | ✓ | ✓ |  |  |
| Bowel movement urgency  during urination | ✓ |  |  |  |  |  |  |  |  |  |
| Constipation | ✓ |  | ✓ |  |  | ✓ |  |  |  | 5 (25) |
| Cramp/pain in abdominal | ✓ |  |  |  |  |  | ✓ | ✓ |  | 1 (5) |
| Cramp/pain in pelvis | ✓ |  |  |  |  |  | ✓ | ✓ |  |  |
| Cramp/pain in rectum | ✓ |  |  |  |  |  |  | ✓ |  | 1 (5) |
| Diarrhea/loose or liquid  bowel movement or  stool/watery   bowel movement |  |  | ✓ |  |  |  | ✓ | ✓ |  | 7 (35) |
| Frequent bowel movement | ✓ |  |  |  |  |  |  | ✓ |  |  |
| Good appetite |  | ✓ |  | ✓ |  |  |  |  |  |  |
| Hemorrhoids |  |  |  |  |  |  |  |  |  | 2 (10) |
| Increase in appetite |  |  |  |  |  |  |  |  |  | 1 (5) |
| Intestinal bleeding |  |  |  |  |  |  |  |  |  | 1 (5) |
| Lacked appetite | ✓ |  | ✓ |  |  |  |  |  |  | 3 (15) |
| Lose control of stool/stool  leakage | ✓ |  |  |  |  |  |  | ✓ |  |  |
| Nausea |  | ✓ | ✓ | ✓ |  |  |  |  |  |  |
| Pain with bowel movement |  |  |  |  |  |  |  | ✓ |  |  |
| Radiation proctitis | ✓ |  |  |  |  |  |  |  |  |  |
| Rectal bleeding |  |  |  |  |  |  |  |  |  | 2 (10) |
| Rectal urgency |  |  |  |  |  |  | ✓ | ✓ |  |  |
| Urgency to have bowel  movement |  |  |  |  |  |  |  | ✓ |  |  |
| Vomiting |  |  | ✓ |  |  |  |  |  |  |  |
| **General symptoms** |  |  |  |  |  |  |  |  |  |  |
| Aches |  | ✓ |  |  |  |  |  |  |  |  |
| Bloated face |  |  |  |  |  |  |  |  |  | 1 (5) |
| Discomfort with face |  |  |  |  |  |  |  |  |  | 1 (5) |
| Dry sinus |  |  |  |  |  |  |  |  |  | 1 (5) |
| Face feeling burning/hot |  |  |  |  |  |  |  |  |  | 2 (10) |
| Feel ill | ✓ | ✓ |  |  |  |  |  |  |  |  |
| Fever |  |  |  |  |  |  |  |  |  | 1 (5) |
| Heavy feeling |  |  |  |  |  |  |  |  |  | 2 (10) |
| Inability to lose weight | ✓ |  |  |  |  |  |  |  |  |  |
| Pain (general) |  | ✓ | ✓ | ✓ |  | ✓ |  |  | 1 (20) | 9 (45) |
| Pain from the waist down | ✓ |  |  |  |  |  |  |  |  |  |
| Polyp |  |  |  |  |  |  |  |  |  | 1 (5) |
| Thirsty |  |  |  |  |  |  |  |  |  | 1 (5) |
| Urge to eat | ✓ |  |  |  |  |  |  |  |  |  |
| Weakness from the waist  down | ✓ |  |  |  |  |  |  |  |  |  |
| Weight change |  |  |  |  |  |  |  | ✓ |  | 12 (60) |
| Weight gain | ✓ |  | ✓ |  |  |  |  |  |  | 5 (25) |
| Weight loss | ✓ | ✓ | ✓ | ✓ |  |  |  |  |  | 7 (35) |
| **Hormonal symptoms** |  |  |  |  |  |  |  |  |  |  |
| Breast pain |  |  |  |  |  |  |  |  |  | 1 (5) |
| Breast tenderness | ✓ |  |  |  |  |  |  | ✓ |  |  |
| Enlarged nipples/breast | ✓ |  | ✓ |  |  |  |  | ✓ |  | 5 (25) |
| Hot flashes/flushes | ✓ |  | ✓ |  |  |  |  | ✓ | 1 (20) | 7 (35) |
| Nipple pain/discomfort |  |  |  |  |  |  |  |  |  | 4 (20) |
| Sore nipples/breast |  |  | ✓ |  |  |  |  |  |  | 5/25 |
| Sweating |  |  |  |  |  |  |  |  |  | 9 (45) |
| **Musculoskeletal Symptoms** |  |  |  |  |  |  |  |  |  |  |
| Bone fractures |  |  |  |  |  |  |  |  |  | 3 (15) |
| Bone pain |  |  |  | ✓ |  |  |  |  |  |  |
| Leg cramp |  |  |  |  |  |  |  |  |  | 2 (10) |
| Leg numbness |  |  |  |  |  |  |  |  |  | 1 (5) |
| Leg pain |  |  |  |  |  |  |  |  |  | 2 (10) |
| Legs feel hot |  |  |  |  |  |  |  |  |  | 1 (5) |
| Loss of bone strength |  |  |  |  |  |  |  |  |  | 1 (5) |
| Loss of muscle mass | ✓ |  |  |  |  |  |  |  | 1 (20) |  |
| Swelling in ankle |  |  | ✓ |  |  |  |  |  |  | 2 (10) |
| Swelling in legs |  |  | ✓ |  |  |  |  |  |  | 2 (10) |
| Weakness in legs |  |  |  | ✓ |  |  |  |  |  | 6 (30) |
| **Sexual function** |  |  |  |  |  |  |  |  |  |  |
| Ejaculation problem |  |  | ✓ |  |  |  |  |  |  |  |
| Erectile dysfunction/have or  maintain erection | ✓ | ✓ | ✓ |  |  |  | ✓ | ✓ |  | 1 (5) |
| Loss of libido | ✓ |  |  |  |  |  |  |  | 1 (20) |  |
| Painful ejaculation | ✓ |  |  |  |  |  |  |  |  |  |
| Reach orgasm | ✓ |  |  |  |  |  | ✓ | ✓ |  |  |
| Sexual desire | ✓ |  |  |  |  |  | ✓ | ✓ | 1 (20) |  |
| Sexual functioning (general) |  |  |  |  |  |  |  |  |  | 4 (20) |
| **Urinary symptoms** |  |  |  |  |  |  |  |  |  |  |
| Blood in semen |  |  |  |  |  |  |  |  |  | 1 (5) |
| Blood in urine | ✓ |  |  |  |  |  |  | ✓ |  | 5 (25) |
| Burning with urination | ✓ |  |  |  |  |  |  | ✓ |  |  |
| Difficulty stopping urine  stream |  |  |  |  |  |  |  |  |  | 1 (5) |
| Difficulty urinating | ✓ | ✓ |  | ✓ |  |  |  |  | 1 (20) | 5 (25) |
| Doesn't feel urination | ✓ |  |  |  |  |  |  |  |  |  |
| Enlarged prostate |  |  |  |  |  |  |  |  |  | 3 (15) |
| Groin pain | ✓ |  |  |  |  |  |  |  |  |  |
| Hard areas on prostate |  |  |  |  |  |  |  |  |  | 1 (5) |
| Incomplete emptying | ✓ |  |  |  |  |  |  | ✓ |  |  |
| Incontinence | ✓ |  |  |  |  |  |  |  | 1 (20) | 1 (5) |
| Loss of penis length | ✓ |  |  |  |  |  |  |  |  |  |
| Narrow urethra |  |  |  |  |  |  |  |  |  | 2 (10) |
| Pain with urination | ✓ |  | ✓ |  |  |  |  | ✓ |  | 3 (15) |
| Pressure/blockage while  urinating | ✓ |  |  |  |  |  |  |  |  |  |
| Residual urine |  |  |  |  |  |  |  |  |  | 3 (15) |
| Urgency to pass urine/  urinary  urgency | ✓ |  | ✓ |  |  |  |  |  | 1 (20) | 3 (15) |
| Urinary control |  |  |  |  |  |  | ✓ | ✓ |  |  |
| Urinary problems |  |  |  |  |  | ✓ |  |  | 3 (60) |  |
| Urinate frequently | ✓ | ✓ | ✓ |  |  |  |  | ✓ | 2 (40) | 14 (70) |
| Urine dripping |  |  |  |  |  |  | ✓ | ✓ |  |  |
| Urine leakage | ✓ |  | ✓ |  |  |  |  | ✓ | 1 (20) | 7 (35) |
| Wake up to urinate /  nocturia | ✓ |  |  |  |  |  |  | ✓ |  | 13 (65) |
| Weak urine stream | ✓ |  |  |  |  |  |  | ✓ |  | 9 (45) |
| **Other** |  |  |  |  |  |  |  |  |  |  |
| Anemia |  |  |  |  |  |  |  |  |  | 1 (5) |
| Aortic aneurysm |  |  |  |  |  |  |  |  |  | 1 (5) |
| Arrhythmia |  |  |  |  |  |  |  |  |  | 1 (5) |
| Bothered by side effects |  | ✓ |  | ✓ |  |  |  |  |  |  |
| High PSA levels (marker at diagnosis) |  |  |  |  |  |  |  |  |  | 18 (90) |
| Increase in PSA |  |  |  |  |  |  |  |  |  | 15 (75) |
| Loss of hair | ✓ |  |  |  |  |  |  | ✓ |  | 5 (25) |
| Neurological disorder |  |  |  |  |  |  |  |  | 1 (20) |  |
| Presence of mass |  |  |  |  |  |  |  |  |  | 1 (5) |
| Rash |  |  |  |  |  |  |  |  |  | 1 (5) |
| Short of breath |  |  | ✓ |  |  |  |  |  |  |  |
