# Supplementary material for: Conceptual assessment of HRQOL among Japanese non‐metastatic castration‐resistant prostate cancer (nmCRPC) patients
Source: Cancer Med. 2022 Jun 30;12(2):1762–78. doi: 10.1002/cam4.4955 (PMC9883429; doi:10.1002/cam4.4955)
Supplement: Supplementary file 5 — Table S5: [file CAM4-12-1762-s001.docx]

**Supplemental Table 5. Gap Analysis for Japanese nmCRPC patients - Impacts**

| **Concepts** | **Literature**  **Review** | **Presence in Prostate Cancer-Specific PRO Instruments** | | | | | | | **Physician Interviews** | **Patient Interviews** |
| --- | --- | --- | --- | --- | --- | --- | --- | --- | --- | --- |
|  |  | **FACT-G + P** | **EORTC QLQ-C30 + PR25** | **NCCN-FACT FPSI-17** | **PC-QoL** | **PROSQOLI** | **UCLA-PCI** | **EPIC** | (N=5),  n (%) | (N=20),  n (%) |
| **Activity Limitation** |  |  |  |  |  |  |  |  |  |  |
| Daily activities |  |  | ✓ |  |  |  |  |  | 2 (40) | 4 (20) |
| Dependent on others |  |  |  |  |  |  |  |  |  | 2 (10) |
| Dressing self |  |  | ✓ |  |  |  |  |  |  |  |
| Eating |  |  | ✓ |  |  |  |  |  |  |  |
| Hobbies |  |  | ✓ |  |  |  |  |  |  |  |
| Leisure activities |  |  | ✓ |  |  |  | ✓ |  |  |  |
| Lifestyle changes |  |  |  |  |  |  |  |  |  | 5 (25) |
| Loss of jobs or business  relationships |  |  |  |  |  |  |  |  |  | 2 (10) |
| Interference with daily  activities | ✓ |  |  |  |  |  |  |  | 2 (40) |  |
| Using toilet |  |  | ✓ |  |  |  |  |  |  |  |
| Usual activities |  |  | ✓ |  |  |  |  |  |  |  |
| Washing self |  |  | ✓ |  |  |  |  |  |  |  |
| Work | ✓ | ✓ | ✓ |  |  |  |  |  | 2 (40) | 1 (5) |
| **Emotional/ Psychological** |  |  |  |  |  |  |  |  |  |  |
| Accepts illness |  | ✓ |  |  |  |  |  |  |  | 4 (20) |
| Afraid of cancer |  |  |  |  |  |  |  |  |  | 1 (5) |
| Afraid of having accident |  |  |  | ✓ |  |  |  |  |  |  |
| Afraid of making mess |  |  |  | ✓ |  |  |  |  |  |  |
| Anxiety/Anxious | ✓ |  |  |  |  |  |  |  | 3 (60) | 10 (50) |
| Avoid situations (if not able  to make it bathroom) |  |  |  | ✓ |  |  |  |  |  |  |
| Concerned |  |  |  |  |  |  |  |  |  | 7 (35) |
| Concern of treatment  effectiveness |  |  |  |  |  |  |  |  | 1 (20) |  |
| Choose other treatment |  |  |  | ✓ |  |  |  |  |  |  |
| Confident cancer is under  control |  |  |  | ✓ |  |  |  |  |  |  |
| Coping made me a stronger  person |  |  |  | ✓ |  |  |  |  |  |  |
| Decreased coping ability | ✓ |  |  |  |  |  |  |  |  |  |
| Depressed | ✓ |  | ✓ |  |  | ✓ |  | ✓ | 4 (80) | 2 (10) |
| Diagnosis shock/surprise |  |  |  |  |  |  |  |  |  | 13 (65) |
| Difficulty concentrating | ✓ |  | ✓ |  |  |  |  |  |  |  |
| Difficulty remembering |  |  | ✓ |  |  |  |  |  |  |  |
| Disappointed |  |  |  |  |  |  |  |  |  | 1 (5) |
| Disbelief at diagnosis |  |  |  |  |  |  |  |  |  | 2 (10) |
| Distress due to bowel  movement |  |  |  |  |  | ✓ |  |  |  |  |
| Distrust in doctor | ✓ |  |  |  |  |  |  |  |  |  |
| Doctor and patient  communication |  |  |  |  |  |  |  |  | 3 (60) | 9 (45) |
| Doctor communicated effects  of treatment |  |  |  | ✓ |  |  |  |  |  |  |
| Embarrassed | ✓ |  |  |  | ✓ |  |  |  |  | 3 (15) |
| Enjoy life |  | ✓ | ✓ |  |  |  |  |  |  |  |
| Enjoy things |  | ✓ |  | ✓ |  |  |  |  |  |  |
| Enough time to make  decision about treatment |  |  |  | ✓ |  |  |  |  |  |  |
| Family concern over illness |  |  |  |  |  |  |  |  | 1 (20) |  |
| Feel beaten up/defeated |  |  |  |  |  |  |  |  |  | 1 (5) |
| Feel dirty |  |  |  | ✓ |  |  |  |  |  |  |
| Feel helpless |  |  |  | ✓ |  |  |  |  |  |  |
| Feel like a man | ✓ | ✓ |  |  |  |  |  | ✓ |  |  |
| Feel like odd man when I  hear talk about sex |  |  |  | ✓ |  |  |  |  |  |  |
| Feel lonely | ✓ |  |  |  |  |  |  |  |  |  |
| Feel other thinks I'm not the man I used to be |  |  |  | ✓ |  |  |  |  |  |  |
| Feel spouse/partner wants to turn to others for affection |  |  |  | ✓ |  |  |  |  |  |  |
| Feel tense |  |  | ✓ |  |  |  |  |  |  |  |
| Feel too emotional |  |  |  | ✓ |  |  |  |  |  |  |
| Feel treatment is wrong for me |  |  |  | ✓ |  |  |  |  |  |  |
| Feel weak/small |  |  |  | ✓ |  |  |  |  |  |  |
| Feeling of loss of control | ✓ |  |  |  |  |  |  |  |  |  |
| Frustration | ✓ |  |  |  |  |  |  |  |  | 3 (15) |
| Hard to think things coolly  an logically |  |  |  | ✓ |  |  |  |  |  |  |
| Health could turn worse |  |  |  | ✓ |  |  |  |  |  |  |
| Hesitant to go out |  |  |  |  |  |  |  |  |  | 2 (10) |
| Humiliated |  |  |  | ✓ |  |  |  |  |  |  |
| Information on treatment |  |  |  | ✓ |  |  |  |  |  |  |
| Irritable |  |  | ✓ |  |  |  |  |  |  | 1 (5) |
| Keep close track of PSA |  |  |  | ✓ |  |  |  |  |  |  |
| Knew right questions to ask  doctor |  |  |  | ✓ |  |  |  |  |  |  |
| Knowing PSA level comforts  me |  |  |  | ✓ |  |  |  |  |  |  |
| Lack of confidence in doctor | ✓ |  |  |  |  |  |  |  |  |  |
| Live in fear that PSA will rise |  |  |  | ✓ |  |  |  |  |  |  |
| Lose hope / hopelessness | ✓ | ✓ |  |  |  |  |  |  |  | 2 (10) |
| Loss of masculinity | ✓ |  |  |  |  |  |  |  |  |  |
| Low self-esteem | ✓ |  |  |  |  |  |  |  |  |  |
| Mood |  |  |  |  |  | ✓ |  |  |  |  |
| Nervous | ✓ | ✓ |  |  |  |  |  |  |  |  |
| Nervous when I don't know where bathroom are |  |  |  |  | ✓ |  |  |  |  |  |
| Not the man I used to be |  |  |  |  | ✓ |  |  |  |  |  |
| No will to keep going |  |  |  |  |  |  |  |  |  | 1 (5) |
| Outlook on life |  |  |  |  | ✓ |  |  |  |  |  |
| Partner avoids embrace, kissing, or caressing |  |  |  |  | ✓ |  |  |  |  |  |
| Patient dissatisfaction (i.e.,  treatment, care, doctor, etc.) |  |  |  |  |  |  |  |  |  | 3 (15) |
| Planning for future |  |  |  |  |  |  |  |  | 2 (40) | 5 (25) |
| Regret |  |  |  |  |  |  |  |  |  | 6 (30) |
| Sad |  | ✓ |  |  |  |  |  |  |  |  |
| Satisfaction of coping with illness |  | ✓ |  |  |  |  |  |  |  |  |
| Satisfaction of present comfort level |  | ✓ |  |  |  |  |  |  |  |  |
| Satisfaction with treatment |  |  |  |  | ✓ |  |  | ✓ |  |  |
| Self-conscious |  |  |  |  |  |  |  |  |  | 3 (15) |
| Shock at symptom |  |  |  |  |  |  |  |  |  | 1 (5) |
| Spouse/partner seems cool and distant |  |  |  |  | ✓ |  |  |  |  |  |
| Stigma around the illness |  |  |  |  |  |  |  |  |  | 2 (10) |
| Thoughts on death and  mortality | ✓ |  |  |  |  |  |  |  |  | 4 (20) |
| Uneasy about present state of  health |  |  |  |  | ✓ |  |  |  |  |  |
| Uneasy thinking about sex life |  |  |  |  | ✓ |  |  |  |  |  |
| Urinary problem complicates everything |  |  |  |  | ✓ |  |  |  |  |  |
| Wish I could change my mind about the treatment |  |  |  |  | ✓ |  |  |  |  |  |
| Wonder if better off with different treatment |  |  |  |  | ✓ |  |  |  |  |  |
| Wonder if treatment worked |  |  |  |  | ✓ |  |  |  |  |  |
| Wonder if worthwhile being treated at all |  |  |  |  | ✓ |  |  |  |  |  |
| Work is fulfilling |  | ✓ | ✓ |  |  |  |  |  |  |  |
| Worried of embarrassment if I try to have sex | ✓ |  |  |  | ✓ |  |  |  |  |  |
| Worry (all types) |  | ✓ | ✓ | ✓ | ✓ |  |  |  | 4 (80) | 13 (65) |
| **Gastrointestinal impacts** |  |  |  |  |  |  |  |  |  |  |
| Not absorbing nutrients | ✓ |  |  |  |  |  |  |  |  |  |
| Plan for bowel movement | ✓ |  |  |  |  |  |  |  |  |  |
| **Physical functioning** |  |  |  |  |  |  |  |  |  |  |
| Change in diet |  |  |  |  |  |  |  |  |  | 8 (40) |
| Changes in weight |  |  |  |  |  |  |  |  |  | 10 (50) |
| Difficulty sleeping due to  frequent urination |  |  | ✓ |  |  |  |  |  |  | 7 (35) |
| Difficulty traveling |  |  |  |  |  |  |  |  |  | 2 (10) |
| Loss of sleep |  |  |  |  |  |  |  |  |  | 10 (50) |
| Meeting needs of family due  to physical condition |  | ✓ |  |  |  |  |  |  |  |  |
| Move body |  |  |  |  |  | ✓ |  |  |  |  |
| Pain interference | ✓ |  | ✓ |  |  |  |  |  |  |  |
| Physical functioning  (general) |  |  |  |  |  |  |  |  |  | 2 (10) |
| Sleep | ✓ | ✓ | ✓ | ✓ |  |  |  |  |  |  |
| Spend time in bed |  | ✓ | ✓ |  |  |  |  |  |  |  |
| Stay in chair |  |  | ✓ |  |  |  |  |  |  |  |
| Strenuous activities |  |  | ✓ |  |  |  |  |  |  |  |
| Unable to travel |  |  |  |  |  |  |  |  |  | 1 (5) |
| Urinate limit activities | ✓ | ✓ |  |  |  |  |  |  |  |  |
| Walking |  | ✓ | ✓ |  |  |  |  |  |  | 8 (40) |
| **Social functioning** |  |  |  |  |  |  |  |  |  |  |
| Become homebody | ✓ |  |  |  |  |  |  |  |  |  |
| Become private | ✓ |  |  |  |  |  |  |  |  |  |
| Difficulty leaving house  (need to be close to a toilet) |  |  | ✓ |  |  |  |  |  |  |  |
| Enjoy time with friends and  family |  |  |  |  |  | ✓ |  |  |  |  |
| Family accepts illness |  | ✓ |  |  |  |  |  |  |  |  |
| Family life interference |  |  | ✓ |  |  |  |  |  |  |  |
| Feel close to friends |  | ✓ |  |  |  |  |  |  |  |  |
| Feel close to partner/support |  | ✓ |  |  |  |  |  |  |  |  |
| Get emotional support from family |  | ✓ |  |  |  |  |  |  |  |  |
| Personal/family relationships |  |  |  |  |  |  |  |  |  | 3 (15) |
| Satisfaction of family  communication about illness |  | ✓ |  |  |  |  |  |  |  |  |
| Social activity interference |  |  | ✓ |  |  |  |  |  |  |  |
| Social relationships |  |  |  |  |  |  |  |  |  | 2 (10) |
| Stress/conflict with partner,  friends, family | ✓ |  |  |  |  |  |  |  |  |  |
| Support from friends |  | ✓ |  |  |  |  |  |  |  |  |
| Unable to sustain relationship | ✓ |  |  |  |  |  |  |  |  |  |
| Unable to sustain social life | ✓ |  |  |  |  |  |  |  |  |  |
| **Sexual Functioning** |  |  |  |  |  |  |  |  |  |  |
| Awakened by erection |  |  |  |  |  | ✓ | ✓ |  |  |  |
| Confident in sexual ability |  |  |  | ✓ |  |  |  |  |  |  |
| Enjoy physical intimacy |  |  |  | ✓ |  |  |  |  |  |  |
| Feel good about sexuality |  |  |  | ✓ |  |  |  |  |  |  |
| Feel good about the way I deal with my own sexual needs and desires |  |  |  | ✓ |  |  |  |  |  |  |
| Have to be careful not to start something that I can finish (physically/sexually) |  |  |  | ✓ |  |  |  |  |  |  |
| Interest in sex |  | ✓ |  |  |  |  |  |  |  |  |
| Satisfaction with sex life |  | ✓ | ✓ |  |  |  |  |  |  |  |
| Sex enjoyable |  | ✓ |  |  |  |  |  |  |  |  |
| Sex feels unnatural |  |  |  | ✓ |  |  |  |  |  |  |
| Sex is too complicated |  |  |  | ✓ |  |  |  |  |  |  |
| Sexual desire |  |  |  |  |  | ✓ | ✓ |  |  |  |
| Sexual functioning (general) |  |  |  |  |  | ✓ | ✓ |  |  |  |
| Sexual intercourse |  |  |  |  |  |  | ✓ |  |  |  |
| Sexually active |  | ✓ |  |  |  |  | ✓ |  |  |  |
| Uncomfortable about being sexually intimate |  | ✓ |  |  |  |  |  |  |  |  |
| Urinary leakage interfere with sexual activity |  |  |  |  |  | ✓ |  |  |  |  |
| Vaginal/anal intercourse (frequency) |  |  |  |  |  | ✓ |  |  |  |  |
| **Urinary impacts** |  |  |  |  |  |  |  |  |  |  |
| Incontinence aid problem |  |  | ✓ |  |  |  |  |  |  |  |
| Incontinence aid to control  leakage |  |  |  |  |  |  | ✓ | ✓ |  |  |
| Plan for urinary frequency | ✓ |  |  |  |  |  |  |  |  | 8 (40) |
| Urinary function |  |  |  |  |  |  | ✓ | ✓ |  |  |
| Wetting pants |  |  |  |  |  |  | ✓ |  |  |  |
| **Other impacts** |  |  |  |  |  |  |  |  |  |  |
| Commuting burden |  |  |  |  |  |  |  |  |  | 4 (20) |
| Financial difficulties | ✓ |  | ✓ |  |  |  |  |  |  | 7 (35) |
| Hospital admittance |  |  |  |  |  |  |  |  | 1 (20) |  |
| Increased treatment cost due  to changed treatment  frequency |  |  |  |  |  |  |  |  |  | 1 (5) |
| Stress (general) |  |  |  |  |  |  |  |  | 2 (40) |  |
| Travel to treatment burden |  |  |  |  |  |  |  |  |  | 2 (10) |
| Overall health/well-being |  |  | ✓ |  |  | ✓ |  |  |  |  |
| Quality of life (general) |  | ✓ | ✓ | ✓ |  |  |  |  |  |  |
|  |  |  |  |  |  |  |  |  |  |  |
